# Corporate Harm Minimisation: Promises and Perils: Comment on "Understanding Marketing Responses to a Tax on Sugary Drinks: A Qualitative Interview Study in the United Kingdom, 2019"

**DOI:** 10.34172/ijhpm.2022.7649

**Published:** 2023-01-22

**Authors:** Jennifer Lacy-Nichols

**Affiliations:** Melbourne School of Population and Global Health, University of Melbourne, Melbourne, VIC, Australia.

**Keywords:** Reformulation, Corporate Power, Commercial Determinants, Corporate Political Activity, Public Health

## Abstract

Taxes on sugary drinks are often used to encourage companies to reformulate their products to reduce the sugar content. This comment discusses how product reformulation can strengthen the market and political power of the food industry, and questions whether these political risks outweigh the public health benefits. It proposes the term ‘corporate harm minimisation’ to describe the strategic adaptation of a public health harm reduction strategy to align with company or industry goals. It concludes by reflecting on the other ways that corporations influence health beyond the production and marketing of ‘unhealthy commodities,’ and why public health actors must explore other strategies to challenge powerful corporations.

 Taxes on sugary drinks are increasingly commonplace. Their professed aim often is to reduce population level sugar consumption, either by making drinks high in sugar more expensive, or by encouraging sugary drink manufacturers to reformulate their products to reduce the sugar content. Sugary drink taxes also change the market and political environments in which soft drink companies act. They have made the sugar content of beverages a potential risk factor for companies, and they helped to decrease the appeal of sugary drinks – aided through other public health policies and messaging about health harms linked to sugar consumption. Ideally, sugary drink taxes would be part of a more comprehensive set of policies, such as restrictions on marketing or graphic warning labels, all of which are designed to reduce the appeal and accessibility of unhealthy foods, and to make food environments healthier.^1^

 My interest is in the politics of sugar reduction. Through that lens, one of the key takeaways from Forde and colleagues’^2^ analysis is this: sugary drink companies are powerful, and they able to adapt. That is, sugary drink taxes are not necessarily a threat to the soft drink industry.

 Analyses of the UK soft drink industry levy (SDIL) found that the main way that SDIL impacted sugar availability was through reformulation: “overall, volume sales have increased while the sugar content has declined.”^3^ My colleagues and I have written elsewhere about the political benefits that product reformulation offers the food industry.^4,5^ In this comment, I first reflect on how the findings of this study align with and also differ from some of my earlier research on The Coca-Cola Company’s (TCCC’s) reformulation strategy and speculate as to why that is. I then consider the applications of this study for research on other industry sectors, in line with the growing focus on the Commercial Determinants of Health.

## The Many Flavours of Product Reformulation

 The finding that the UK SDIL accelerates marketing activities already taking place is consistent with research of TCCC’s reformulation policies and practices in other countries. For example, in Australia the company is actively reformulating sections of its portfolio, has acquired low- and no-sugar beverages (eg, the country’s leading bottled water brand Mount Franklin), and has launched smaller portion sizes for several of its flagship brands.^4^ Thus far, Australia does not have a tax on sugary drinks, although public health organisations have actively lobbied in support of one, and the 2018 Senate Inquiry into obesity recommended one.^6^ Although these are Australian examples, the impetus for these reformulation activities came from the global parent company in Atlanta, who in 2015 launched a global strategy to reformulate beverages across its portfolio. It is likely therefore, that TCCC is pursuing similar reformulation activities in many countries simultaneously. Future research could take a longitudinal approach to track how different policy developments accelerate or deter reformulation, and whether these are confined to a single country, or whether companies make changes across multiple jurisdictions (such as TCCC has done, albeit unevenly).

 Forde and colleagues’^2^ finding that brands with identities tied to sugar are less likely to reformulate and more likely to reduce portion sizes is somewhat different from our research of TCCC’s reformulation practices. In the public health world, TCCC, and its flagship brand Coca-Cola, is loosely synonymous with sugar. Indeed, the company’s internal reports and transcripts from investor calls make it clear that the company is aware of its reputation as the world’s largest sugary drink manufacturer, and the market and regulatory risks this brings. Yet the company is reformulating many of its products, even within the flagship Coca-Cola brand (which comprises Coca-Cola Classic, Diet Coke, and others).

 Early in my PhD (which analysed the soft drink industry’s response to obesity), I used to joke that if TCCC reformulated its flagship Coca-Cola brand, I’d eat my thesis. In some ways, the joke holds true, but it depends greatly on what is meant by ‘reformulation.’ Despite claims by the company that the original recipe is preserved in a vault in Atlanta (it might be), in practice the Coca-Cola Classic recipe has changed many times (often revealed during the company’s historical court battles). But remove the sugar entirely? I wager my thesis is safe.

 Our research suggests that for sugary drink manufacturers, there are essentially three variables that shape reformulation (Figure). First is whether a new product is developed or the original product is changed. For example, Coke Zero/Coca-Cola Zero Sugar are an alternative to Coca-Cola Classic, whereas in the United Kingdom the original Sprite was reformulated to reduce its sugar content. This leads to the second variable, which is how the recipe is changed. Often, sugar is replaced with a non-caloric sweetener with the aim of maintaining a similar taste and sweetness profile (eg, aspartame, stevia, acesulphame potassium, etc). This approach has raised public health concerns, as research has found that while sugar content may be decreasing in both foods and beverages, artificial sweetener content is increasing, resulting in a food supply that is sweeter and more ultra-processed overall.^7^ However, many companies are slowly reducing the sugar content without replacing the sugar, resulting in a gradually less sweet beverage. To avoid consumer rejection of a rapid change in taste, this strategy may be slower to implement, which also explains why we see so much reformulation activity preceding the SDIL. In short, the soft drink industry has seen the writing on the wall. This leads to the third variable, which is how the reformulation is communicated to consumers. Some reformulation is very public – for example, Coke Zero (now Coca-Cola No Sugar) had a high-profile marketing campaign that presented the product as a no-sugar alternative. Other products are reformulated in ‘stealth’ without any public campaigns.^4^ By engaging in reformulation under the radar, companies are able to incrementally change the recipes of their products without the risk of consumer backlash. TCCC in particular is aware of these risks, as their reformulation of New Coke in 1985 is one of the more infamous examples of consumer backlash.

**Figure:**
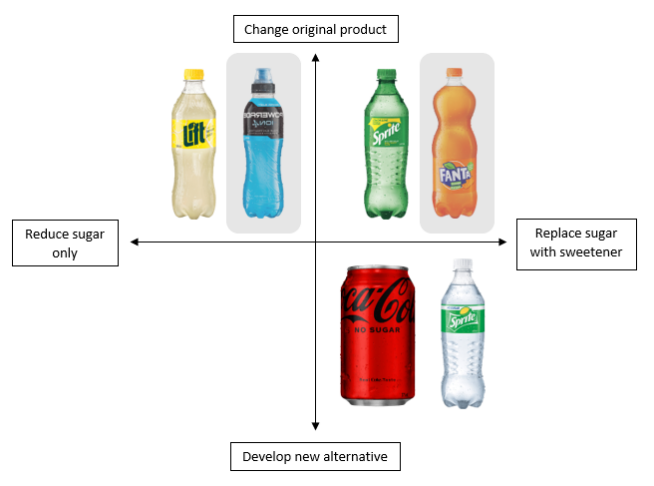
Three Variables Shaping Soft Drink Reformulation: Product, Recipe, Marketing. Grey boxes indicate products with minimal or no launch.

 These different approaches to reformulation may help to explain the finding from Forde and colleagues that brand identities tied to sugar are less likely to reformulate. I suggest it would be more accurate to say that these brands are more likely to pursue some forms of reformulation over others. Future research could compare the reformulation practices of different brands whose identities are more or less tied to sugar to help answer this question.

## Corporate Harm Minimisation: Promises and Perils

 Voluntary product reformulation is a form of what can be termed ‘corporate harm minimisation.’ Corporate harm minimisation can be conceptualised as the strategic adaptation of a public health harm reduction strategy to align with company or industry goals. Other food industry examples include voluntary removal of products from schools, front-of-pack labelling, or nutrition education campaigns. These are all activities that public health actors have recommended.^1^ However, the key difference (and what makes these corporate harm minimisation strategies) is that they are corporate-driven. They lack regulation. They lack accountability. And while they appear to align with public health goals, most fall far short. Corporate harm minimisation strategies are designed on corporate terms and generate significant market and political benefits for the company. These risks require careful consideration, as they may undermine other efforts to hold powerful corporations to account.^8^

 Forde and colleague’s paper presents a richly detailed analysis of a corporate strategy. It does not speculate about the political ramifications of this strategy (which could be an entire study unto itself). Reading between the lines of the data and findings, two points made in their paper highlight the risks of corporate harm minimisation. First, large companies found it easier to adapt to the SDIL. Second, product reformulation benefits companies both politically and financially: “the idea that this was some kind of financial catastrophe has proven very untrue.”^2^ In other words, it appears that product reformulation, especially for large companies, is a profitable and politically savvy strategy. This raises the question: while there may be some health benefits to product reformulation, does the voluntary product reformulation that we see today present more risks than benefits?

 Beyond the potential food quality limitations of reformulation (these have been extensively analysed elsewhere, see for example Scrinis and Monteiro),^9^ there are also political risks. Reformulation activities help companies position themselves as benevolent actors working in the public interest, they facilitate partnerships with governments and health organisations, and they help to decrease political appetite for regulation.^4,8^ They also grow profits and market share, which can be used to engage in other practices that protect the corporate bottom line at the expense of public health.^10^ Perhaps most importantly, reformulation alone does not change the wider system characterised by unequal power dynamics between multinational corporations and public health and civil society actors advocating for change.^11^ I speculate that if these political risks of reformulation were be factored into decisions alongside public health considerations, the perceived benefits of voluntary reformulation would diminish considerably. While this is not the norm for policy makers, we can see some movement towards managing the risks of corporate power and influence. One approach is embedding conflict of interest tools in decision-making. A second approach seeks to increase the participation of civil society and community voices at all levels of government.^12^

 If we think of the broad concept of reformulation as swapping out a part but keeping the whole intact, this ‘harm minimisation’ strategy can be seen in other industries too. The product reformulation that we see in the sugary drinks industry has clear parallels to reformulation activities within other “unhealthy commodity industries.”^13^ Low- or no-alcohol beverages and e-cigarettes are two examples of products that have had some of the ‘harmful’ elements removed. Whether they offer sufficient public health benefit is subject to considerable debate. We can extend the analogy to corporate responses to sustainability issues as well. Many automobile companies have developed hybrid or electric cars, and oil and gas companies are investing in more renewable energy sources. While certainly part of the transition to more sustainable energy sources, they are also strategic efforts to de-risk the portfolios of these corporations. And, just like the sugary drink industry, the ‘less harmful’ alternatives are profitable. Although the ‘people-planet-profits’ rhetoric is politically seductive, it is also risky. These profits can be used to continue other, harmful corporate practices, whether that is the continued production of unhealthy or unsustainable products in other jurisdictions or lobbying to avoid industry regulations. To avoid such loopholes, more comprehensive regulations governing corporate practices are required, such as the United Nations Human Rights Council’s draft instrument to regulate transnational corporations.

## From Corporate Products to Corporate Practices

 If we move beyond a focus on reformulating specific ingredients and commodities, what could we change? The premise of reformulation is to tweak a component of a product to make it less harmful. It does not necessarily change who owns the product or the system that enables its production and use. The focus on specific commodities can distract from other ways that corporations influence health, whether through their employment practices, their supply chains, the political strategies to block health policies, or their tax avoidance that effectively defunds the public sector. These activities are important determinants of health (see elsewhere for discussion of the commercial determinants of health).^10,14^

 If we truly want to progress towards food systems that are healthy and fair, transformative actions will be required that challenge the political power and influence of the corporations that currently dominate the industrial food system. Sugary drink taxes (and the product reformulation they encourage), must be coupled with other policies and approaches that address power disparities (wealth or solidary taxes are one of many possibilities).^15^

## Ethical issues

 Not applicable.

## Competing interests

 Author is a recipient of a fellowship from the Victorian Health Promotion Foundation.

## Author’s contribution

 JLN is the single author of the paper.
